# Role of Tuber Developmental Processes in Response of Potato to High Temperature and Elevated CO_2_

**DOI:** 10.3390/plants10050871

**Published:** 2021-04-26

**Authors:** Chien-Teh Chen, Tim L. Setter

**Affiliations:** 1 Department of Agronomy, National Chung Hsing University, Taichung 402, Taiwan; ctchen41@dragon.nchu.edu.tw; 2 Section of Soil and Crop Sciences, School of Integrative Plant Science, Cornell University, Ithaca, NY 14853, USA

**Keywords:** *Solanum tuberosum*, tuber, sink organ, ambient temperature, cell proliferation

## Abstract

Potato is adapted to cool environments, and there is concern that its performance may be diminished considerably due to global warming and more frequent episodes of heat stress. Our objectives were to determine the response of potato plants to elevated CO_2_ (700 μmol/mol) and high temperature (35/25 °C) at tuber initiation and tuber bulking, and to elucidate effects on sink developmental processes. Potato plants were grown in controlled environments with treatments at: Tuber initiation (TI), during the first two weeks after initiating short-day photoperiods, and Tuber bulking (TB). At TI, and 25 °C, elevated CO_2_ increased tuber growth rate, while leaves and stems were not affected. Whole-plant dry matter accumulation rate, was inhibited by high temperature about twice as much at TI than at TB. Elevated CO_2_ partially ameliorated high temperature inhibition of sink organs. At TI, with 25 °C, elevated CO_2_ primarily affected tuber cell proliferation. In contrast, tuber cell volume and endoreduplication were unaffected. These findings indicate that the TI stage and cell division is particularly responsive to elevated CO_2_ and high temperature stress, supporting the view that attention should be paid to the timing of high-temperature stress episodes with respect to this stage.

## 1. Introduction

Atmospheric CO_2_ levels and average global temperature have risen in the last decades and earth air temperature is predicted to continue to increase as a result of the rise in the levels of CO_2_ and other greenhouse gases [1,2]. While it is recognized that these trends will impact crop production and food security [3], crop growth models of potato (*Solanum tuberosum*) response to environmental variables, suggest that our understanding of the effects of elevated atmospheric CO_2_ and temperature remains far from complete and in need of improvement [4]. Findings from eight global climate models that were used to simulate potato agronomic and climate responses indicate that model variation was modest for predicted CO_2_ but was particularly uncertain for temperature [5]. Furthermore, climate predictions indicate that accompanying an increase in global average temperatures of 1.5 °C, will be a sharp rise in the likelihood of extreme heat stress events [6].

Atmospheric CO_2_ enrichment can affect potato growth and productivity by directly increasing leaf photosynthesis, in accordance with leaf photosynthetic CO_2_ response, and also by eliciting partial stomatal closing and increasing water use efficiency [7,8,9]. In addition, altered rates of photosynthate availability can modify partitioning of photosynthate among plant parts, which in turn can have feedback effects on photosynthesis [10,11,12]. These effects may lessen the potential benefit of elevated CO_2_ on potato photosynthesis, as has been found in season-long free air CO_2_ enrichment (FACE) [11,13,14]. In potato, elevated CO_2_ can lead to greater stimulation of tuber development than above-ground plant parts such that harvest index is increased [15].

Consistent with its Andean origin, potato is a heat-sensitive crop, which performs best at relatively cool temperatures [3]. For example, tuber dry matter partitioning in the temperate-adapted cultivar Katahdin was decreased 65% when day/night temperature was increased from 24/15 °C (max/min) to 30/21 °C [16]. Increases in temperature have the potential of affecting growth and development in several ways. High temperature inhibits photosynthesis in potato, as is generally the case in cool-adapted C3 plants [17]. In support of this explanation, some studies have reported that above-ground temperature has a greater effect than either root or stolon temperature on tuber growth [18]. Moreover, studies to determine the cause of poor yield of heat-susceptible potato genotypes grown at high temperature suggest it is related to inhibited photosynthesis and insufficient availability of the transportable sugar, sucrose [19]. In addition, warm temperature can have direct effects that inhibit tuber initiation and growth [20,21], thereby diverting photosynthate from tubers to shoots [15,22,23]. High temperature in the tuber zone inhibits tuber development [24], and studies have indicated that below-ground high temperature has greater effects than above-ground temperature due to inhibition of tuber development and sink strength, which shifts partitioning to above-ground shoots [25]. Studies have indicated that the negative effects of high temperature on potato growth and yield is dependent on the growth stage and is especially great at the early stages of tuber growth [26].

Potato is adapted to relatively cool growing environments, and it has been predicted that its yield will diminish due to global warming and more frequent episodes of heat stress [3]. It is possible that elevated atmospheric CO_2_ concentration, may partially ameliorate negative effects of heat stress by enhancing photosynthesis and lessening negative effects on potato tuber growth. Our objectives were to determine the response of potato plants to elevated CO_2_ and high temperature treatment at tuber initiation and tuber bulking, and to elucidate effects on sink developmental processes, including tuber cell division and expansion, levels of sugars, and certain carbohydrate-metabolizing enzymes. Our findings indicate that cell proliferation is particularly responsive to elevated CO_2_ and high temperature treatment, and that elevated CO_2_ partially ameliorates the effects of high temperature.

## 2. Results

### 2.1. The Effects of High Temperature and Elevated CO_2_ on Potato Plant Growth

#### 2.1.1. Tuber Initiation Stage

**Figure 1:**
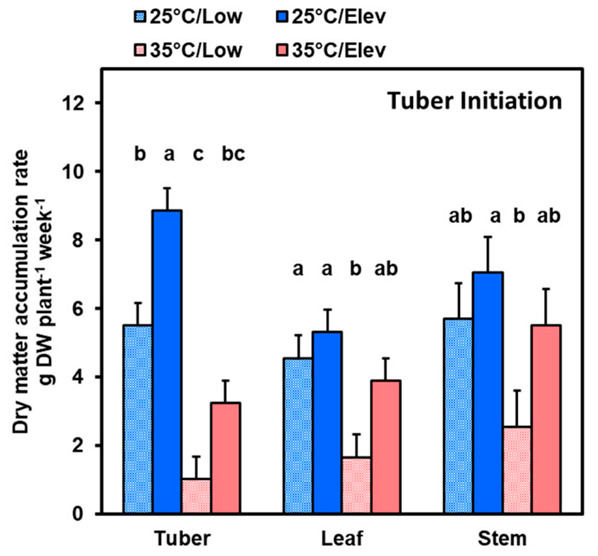
Tuber, leaf, and stem dry matter accumulation rate at the tuber initiation stage in plants that were given temperature (25 or 35 °C) and CO_2_ (Low_CO_2__ or Elev_CO_2__) treatments during the 2-week tuber initiation period. To initiate tuberization, plants that were grown in long days were transferred to short-day growth chambers and exposed to treatments for 2 weeks, then harvested. Bars represent averages ± SEM of 6 replicates; values labeled with different letters are significantly (*p* ≤ 0.05).

Dry matter accumulation rate (DMAR) during each treatment period was estimated from the difference in dry matter of plants harvested at the onset of a treatment, and those harvested 2 weeks later at the conclusion of the treatment. At the tuber initiation stage, high temperature (35 °C) substantially decreased tuber growth compared to normal temperature (25 °C) in both low (Low_CO_2__) and elevated (Elev_CO_2__) CO_2_ environments (Figure 1). High temperature under Low_CO_2__ decreased tuber DMAR by 82%, and had similar effects (about 60% decrease) on DMAR of leaves and stems. Under Elev_CO_2__, high temperature decreased DMAR somewhat less: by 63% in tubers and by about 25% in leaves and stems. On a whole plant basis (sum of the three plant parts), high temperature decreased DMAR by 56 and 35% in plants at Low_CO_2__ and Elev_CO_2__, respectively (Table 1). In contrast to temperature, elevated CO_2_ provided distinctly greater benefit to tubers compared to other plant parts. Elevated CO_2_ at 25 °C increased DMAR 60% in tubers, but did not affect DMAR in leaves and stems. At 35 °C, elevated CO_2_ appeared to increase DMAR, and thereby partially ameliorate high temperature inhibition, in all three plant parts, but did not achieve statistical significance (*p* ≤ 0.05).

#### 2.1.2. Tuber Bulking Stage

**Figure 2:**
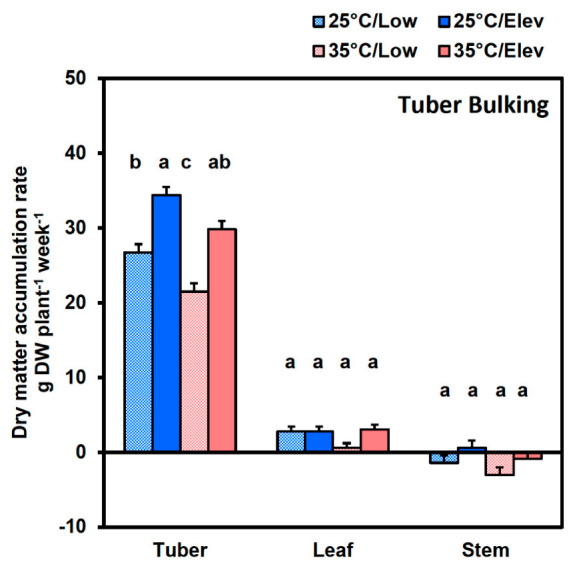
Tuber, leaf, and stem biomass accumulation rate at the tuber bulking stage following temperature (25 or 35 °C) and CO_2_ (Low_CO_2__ or Elev_CO_2__) treatments during the 2 week tuber bulking stage. Plants were given two weeks of shortday conditions at 25 °C with low CO_2_ to induce tuberization, then maintained at short days and exposed to treatments for 2 weeks, and then harvested. Bars represent averages ± SEM of 6 replicates; values labeled with different letters are significantly (*p* ≤ 0.05) different.

At the tuber bulking stage, tuber growth predominated, as there was negligible leaf and stem growth (Figure 2). Elevated CO_2_ increased tuber DMAR about 30 to 40% at both temperatures (Figure 2), and this was reflected in whole plant DMAR (the sum of tuber, leaf and stem) (Table 1). Comparing across growth stages, high temperature had greater effects on whole plant DMAR (Table 1) at the tuber initiation stage than at the tuber bulking stage (overall about −45 and −24%, respectively). In contrast, the effects of elevated CO_2_ on whole-plant DMAR were fairly similar at the two stages. Elevated CO_2_ at 25 °C increased whole plant DMAR by 29% at tuber initiation and by 34% at tuber bulking. Given that whole plant DMAR is a measure of net whole-plant photosynthesis in excess of respiration, this indicates that elevated CO_2_ provided consistent benefit throughout these phases of development. The main difference for CO_2_ effects across development stages was the case with high temperature during tuber initiation, where elevated CO_2_ benefitted leaf and stem growth in addition to tuber growth, whereas only tubers benefitted at the tuber bulking stage. This suggests that growing plant parts have a development window within which they are capable of responding to improved photosynthate supply.

### 2.2. Tuber Numbers and Size

**Figure 3:**
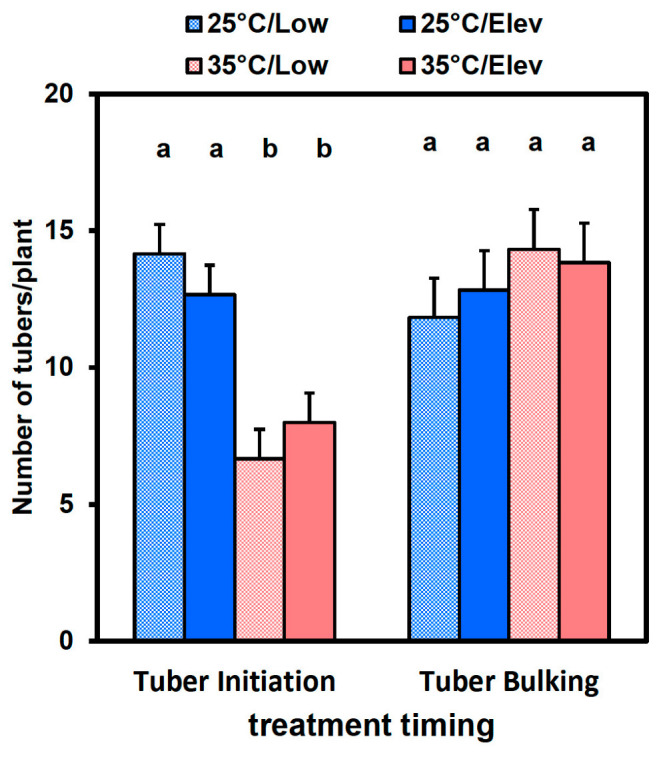
Tuber number in response to temperature (25 or 35 °C) and CO_2_ (Low_CO_2__ or Elev_CO_2__) treatments at stages of tuber initiation and tuber bulking. Plants were harvested at 2 weeks after starting short-day exposure (tuber initiation period), or 4 weeks after starting short-day exposure and receiving temperature and CO_2_ treatments for the latter 2 weeks of the period (tuber bulking period). Tubers exceeding 1 cm diameter were counted. Bars represent averages ± SEM of 6 replicates; values labeled with different letters are significantly (*p* ≤ 0.05) different.

High temperature at the tuber initiation stage substantially decreased the number of tubers per plant (−37% at Low_CO_2__, and −53% at Elev_CO_2__) (Figure 3). Plants receiving treatments during tuber bulking were exposed to 25 °C and Low CO_2_ during their tuber initiation stage, and they did not further increase their tuber numbers during tuber bulking. Hence, there were no treatment effects on tuber numbers at tuber bulking (Figure 3). Elevated CO_2_ did not affect tuber number at either stage. Histograms of the distribution of biomass among individual tubers at tuber initiation and tuber bulking, appeared to show that temperature × CO_2_ treatment interactions affected the proportions of tubers in various size classes (Supplementary Figure S1).

### 2.3. The Effects of High Temperature and Elevated CO_2_ on Potato Tuber Cell Properties

**Figure 4:**
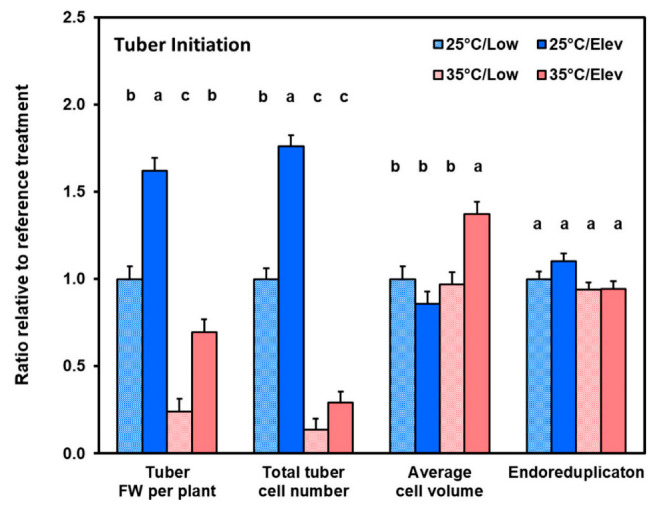
Relative increase in tuber properties with respect to 25 °C/Low_CO_2__ control in response to temperature (25 or 35 °C) or CO_2_ treatments (Low_CO_2__ or Elev_CO_2__) at the tuber initiation stage. Tuber weight and tuber cell number were summed for each plant and represent the increments of tuber fresh-weight growth and cell proliferation during the treatment periods. Data are expressed relative to the 25 °C/Low_CO_2__ reference which was: 80.5 g tuber fresh weight plant^−1^, 1.25 × 10^8^ tuber cells plant^−1^, 7.9 × 10^5^ μm^3^ average tuber cell volume, and 31.3% average proportion of tuber cells in endoreduplication categories (≥ 8C nuclear DNA content). See Materials and Methods for details. Bars represent averages ± SEM of 6 replicates; values labeled with different letters are significantly (*p* ≤ 0.05) different.

**Figure 5:**
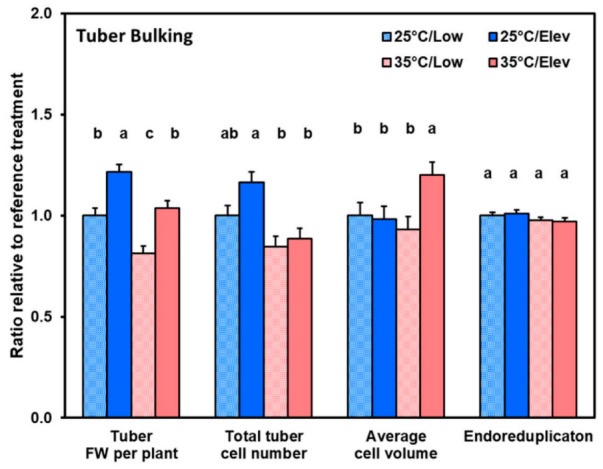
Relative increase in tuber properties with respect to 25 °C/Low_CO_2__ control in response to temperature (25 or 35 °C) or CO_2_ treatments (Low_CO_2__ or Elev_CO_2__) at the tuber bulking stage. Tuber weight, and tuber cell number were summed for each plant and represent the increments of tuber fresh-weight growth and cell proliferation during the treatment periods. Data are expressed relative to the 25 °C/Low_CO_2__ reference which was: 529 g tuber fresh weight plant^−1^, 6.12 × 10^8^ tuber cells plant^−1^, 1.17 × 10^6^ μm^3^ average tuber cell volume, and 48.2% average proportion of tuber cells in endoreduplication categories (≥8C nuclear DNA content). See Materials and Methods for details. Bars represent averages ± SEM of 6 replicates; values labeled with different letters are significantly (*p* ≤ 0.05) different.

Given that plant growth responses to CO_2_ and temperature treatments primarily involved changes in tuber growth, we determined the extent to which each aspect of tuber growth was altered. Three aspects were evaluated: (1) cell division, which we evaluated by counting cell nuclei with flow cytometry; (2) cell expansion growth, which we evaluated by calculating average cell volume; and (3) post-mitotic DNA replication (endoreduplication), which we evaluated from the nuclear DNA analysis as the count of nuclei with DNA content ≥ 8C (where C is the haploid DNA content). The data in Figure 4 and Figure 5 are shown as a proportion of the Low_CO_2__/25 °C control so that treatment effects on various tuber properties can be readily compared. At tuber initiation (Figure 4), high temperature substantially decreased tuber fresh weight, and correspondingly, total tuber cell number per plant. Elevated CO_2_ significantly (*p* ≤ 0.05) increased cell proliferation at 25 °C, while at 35 °C the number of cells produced was very low and effects of elevated CO_2_ could not be discerned (Figure 4). In contrast, cell volume was not affected by CO_2_ and temperature treatments, except at high temperature and elevated CO_2_, where average cell volume was increased. The extent of endoreduplication, which reflects nuclear DNA reduplication in the absence of intervening mitosis, was not affected by treatments. Similarly, at tuber bulking (Figure 5), elevated CO_2_ and high temperature affected cell proliferation in some treatment combinations, and at elevated CO_2_ and temperature cell volume increased; however, the magnitude of treatment effects was less than during tuber initiation.

### 2.4. The Effects of High Temperature and Elevated CO_2_ on Sugar Levels and Invertases

#### 2.4.1. Sugars

**Figure 6:**
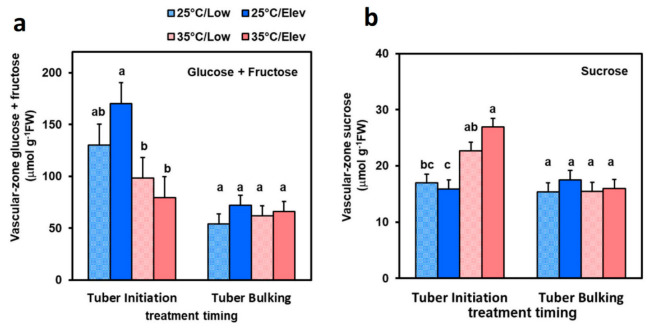
Tuber phloem-rich perimedullary-zone hexose (glucose + fructose) (**a**) and sucrose (**b**) in plants exposed to temperature (25 or 35 °C) or CO_2_ (Low_CO_2__ or Elev_CO_2__) treatments at the tuber initiation stage (left of each figure) or tuber bulking stage (right). Plants were harvested after exposure to treatments for 2 weeks. Bars represent averages ± SEM of 6 replicates; values labeled with different letters are significantly (*p* ≤ 0.05) different.

Given the evidence that cell proliferation in tubers responded substantially to treatments, we considered the hypothesis that CO_2_ and temperature treatments could affect photosynthate supply or consumption in the phloem-rich zone of tubers. Phloem-containing perimedullar-zone tissues were sampled to provide an indication of carbohydrate status in the region of abundant phloem and rapid cell proliferation. At the tuber initiation stage with elevated CO_2_, high temperature treatment decreased hexose (glucose + fructose) concentrations (Figure 6a). A decrease in sugar concentration would be expected if high temperature increases the rate of metabolism and use of sugar. However, high temperature increased sucrose concentration at tuber initiation (Figure 6b), suggesting that high temperature did not have a net effect on sugar import/utilization, but might affect conversion of one form to the other. There were no treatment effects on sugars at the tuber bulking stage. Trends in sugar concentrations were toward higher values in elevated CO_2_, though the differences were not significant (*p* ≤ 0.05). This finding suggests that the conversion of sucrose to hexoses might be affected by the treatments.

#### 2.4.2. Invertases

**Figure 7:**
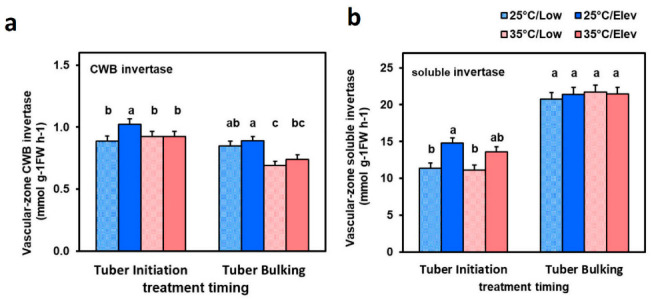
Tuber phloem-rich perimedullary-zone (**a**): cell wall bound (CWB) acid invertase activity and (**b**): soluble acid invertase activity in plants exposed to temperature (25 or 35 °C) or CO_2_ (Low_CO_2__ or Elev_CO_2__) treatments at the tuber initiation stage (left of each figure) or tuber bulking stage (right). Plants were harvested after exposure to treatments for 2 weeks. Bars represent averages ± SEM of 6 replicates; values labeled with different letters are significantly (*p* ≤ 0.05) different.

To evaluate whether treatments affected enzymes for converting imported sucrose into hexoses, we measured cell-wall-bound (CWB) and soluble invertase activities in tubers. Activities of CWB and soluble invertases were analyzed in perimedullary zones where phloem is abundant and is delivering photosynthate to growing tissues. In the elevated CO_2_ treatment at tuber initiation, and at both CO_2_ treatments at tuber bulking, CWB invertase activity was slightly decreased in response to high temperature (Figure 7a). Though slight, the direction of this inhibition is consistent with it playing a role in the observed increase in sucrose and decrease in hexose in response to high temperature (Figure 6a,b). In contrast, at tuber initiation with low temperature, the elevated CO_2_ treatment increased both CWB invertase and soluble invertase activity (Figure 7a,b). The direction of this stimulation of invertase activity is consistent with it playing a role at low temperature for maintaining low sucrose and a tendency for higher hexose in response to elevated CO_2_ (Figure 6).

## 3. Discussion

### 3.1. Elevated Atmospheric CO_2_ Effect on Whole Plant Biomass Accumulation

The current findings indicated that two-week treatments involving elevated atmospheric CO_2_ concentration at 25 °C increased dry matter accumulation rate by about 30%. This is consistent with previous findings involving short-term elevated CO_2_ treatments which have been conducted in growth chambers and controlled environments [27,28]. However, studies involving long-term exposure to elevated CO_2_ in open-top and FACE experiments have indicated that such increases in whole-plant photosynthesis are not generally sustained, such that benefit from CO_2_ enrichment is typically only about half as much in the long term [11,13,14]. Several factors are involved in this phenomenon, among them partial stomatal closing, limitations in nitrogen supply, and acclimation as plants adjust their development of sink organs in response to an incremental increase in photosynthate availability, and feedback inhibition of photosynthesis [7,9,29]. One of the objectives of the current study was to determine the extent to which potato plants respond to short-term elevated CO_2_ by stimulating growth of sink organs: tubers, stems, and expanding leaves. In previous full-season studies of potato exposed to CO_2_ enrichment with open-top chambers, elevated CO_2_ increased tuber growth and yield, and tubers were preferentially favored, as the partitioning ratio of below-ground to above-ground biomass was increased [15]. Similar findings have been reported for experiments in growth chambers where precise control of the timing of treatments can be achieved [30]. Consistent with this, the current study indicated that elevated CO_2_ at 25 °C during tuber initiation increased tuber dry matter accumulation rate (DMAR) by more than 60%, while leaf and stem DMAR was not significantly (*p* ≤ 0.05) affected, even though these organs collectively represented almost two thirds of the sink-organ DMAR (Figure 1). At the tuber bulking phase, when most of plant photosynthate is used for starch storage in tubers, leaves had very low DMAR and stems had a net loss in dry matter, so almost all the benefit from elevated CO_2_ was obtained by tubers.

### 3.2. High Temperature Stress Effect on Whole Plant Biomass Accumulation

Potato grows optimally in cool climates (15 to 22 °C) whereas its growth is inhibited in warm or hot environments [10,21,31]. Heat stress can exert detrimental effects on many growth and development processes, so the current studies, involving 2-week treatments at two discrete developmental stages, provide information on the relative susceptibilities of various developmental processes in tubers. Whole-plant dry matter accumulation rate, a measure of whole plant photosynthesis in excess of respiration, was inhibited by high temperature about twice as much at the stage of tuber initiation than at tuber bulking (Table 1; Figure 1 and Figure 2). Similar findings were obtained in studies of elevated temperature under field conditions with temporary transparent chambers in which temperature was increased by about 4 °C for discrete periods of time [32,33]. Moreover, studies that involved treatments with 35 °C stress, similar to the present investigation, showed that tuber growth was strongly decreased by exposure to 35 °C at early phase of tuberization but had a diminishing effect at later stages [26]. In contrast, in a greenhouse study to test the effect of high temperature (30 °C) before tuber initiation versus after tubers had begun bulking, both treatments reduced tuber growth to a similar extent [34]. However, this experiment involved plants that were exposed starting at the date of planting, prior to exposure to tuber-inducing photoperiods, whereas in the current study, plants were first grown with long days, which is a non-inductive photoperiod, then tuber induction was begun at a discrete experimentally determined time. To separate high temperature effects on above-ground and below-ground plant parts, investigators have used growth chambers with root-zone temperature control [25]. After tuber induction, high temperature whole-plant or below-ground treatments both resulted in a reduction in tuber growth and decreased photosynthetic rates [25]. This suggests that communication of root-zone environment or sink-strength status is conveyed to leaves and this may determine the extent to which plant biomass is diminished. Hence, potato response to high temperature appears to depend on differences in sensitivity of various plant parts and of the developmental processes at different stages.

Amelioration of high temperature effects by Elevated CO_2_. In the current study, the percentage inhibition of dry matter accumulation rate (DMAR) by high temperature was somewhat greater in Low_CO_2__ than in Elev_CO_2__ treatments (56 and 35%, respectively, at tuber initiation; and 32 and 15%, respectively, at tuber bulking), consistent with earlier findings [33]. In several studies, elevated CO_2_ has been found to partly ameliorate high temperature stress [35,36]. Although the 35 °C environment decreased both tuber and whole plant DMAR at both tuber initiation and tuber bulking stages (Figure 1 and Figure 2), the elevated CO_2_ treatment (35 °C/Elev_CO_2__) at tuber initiation eased some of the impact of heat stress so that the DMAR of tubers and whole plants recovered to rates comparable to the normal treatment (25 °C/Low_CO_2__). At tuber bulking, elevated CO_2_ eased most of the heat stress, such that tuber DMAR at 35 °C /Elev_CO_2__ was mid-way between 25 °C/Low_CO_2__ and 25 °C/Elev_CO_2__.

### 3.3. Tuber Growth and Development

#### 3.3.1. High Temperature Effects on Components of Tuber Development

Both tuber cell proliferation and cell expansion (and associated organelle and enzyme development) are needed to develop starch storage capacity for tuber bulking in potato tubers [37]. In previous studies, high temperature suppressed tuber initiation so that tuber numbers per plant were decreased [33,38,39]. Consistent with this, we found that tuber numbers were decreased by high temperature when applied at tuber initiation (Figure 3). However, the lower number of tubers may have been due, in part, to the poorer cell division and expansion growth of tubers at high temperature (Figure 4) such that more of the initiated tubers were smaller than the 1 cm diameter cutoff used for tuber counts. Outcomes similar to this were observed in studies with other treatments that were unfavorable to tuber development and growth, such as shade and long-day photoperiods during tuber bulking [30,40]. Moreover, in the present study photoperiod was switched from 14 to 10 h day length, which provides a very strong tuber inductive signal that may have been sufficient to overcome inhibitory influences due to high temperature [41,42,43,44].

#### 3.3.2. Elevated CO_2_ Effects on Components of Tuber Development

We obtained data on three components of early tuber development: cell proliferation, cell expansion, and post-mitotic DNA endoreduplication (Figure 3 and Figure 4). At the tuber initiation stage, with 25 °C, elevated CO_2_ had a substantial stimulative effect on cell proliferation such that tuber cell numbers in the elevated CO_2_ treatment were 62% greater than with the corresponding low CO_2_ control (Figure 4). In contrast, cell volume and the proportion of cells that had undergone endoreduplication were unaffected. Cell proliferation continued during the tuber bulking stage and cell numbers responded to Elev_CO_2__ in proportion to tuber weight, while average cell volumes and endoreduplication were not affected (Figure 5). These data are in agreement with previous studies which have shown that cell proliferation is highly responsive to elevated CO_2_ while cell expansion growth is less responsive at the early phase of tuber development [30]. Transcriptome studies on potato tubers show that genes associated with cell division are highly upregulated during tuber initiation [45].

#### 3.3.3. Sugar Signaling in Tuber Development

It is plausible that sugar status in tubers serves as a component of signaling pathways for stimulating enhanced growth in the tubers. At both tuber initiation and tuber bulking, high temperature decreased tuber dry matter (Figure 1 and Figure 2) and fresh weight (Figure 4 and Figure 5) growth. The percentage inhibition at each stage was approximately the same as that for respective whole-plant DMAR, so it is plausible that the inhibitory response in tubers involved photosynthate status as a cue. This interpretation is also supported by the approximately similar pattern of CO_2_ benefit in tubers compared with that in whole plants in response to the Elev_CO_2__ treatments, although the magnitude of response was greater in tubers than leaves and stems, as discussed above. Studies have identified sugar signaling in potato tubers that regulate patatin storage-protein synthesis [46] and expression of starch pathway enzymes [25,47,48], which are thought to involve sucrose sensing. Consistent with this, when the levels of Trehalose-6-phosphaste (T6P), a sugar-signaling metabolite, was elevated with tuber-specific overexpression of the T6P synthase, or lowered with expression of T6P phosphatase, expression of genes involved in tuber cell proliferation were affected [47]. Studies have shown linkages between sugar signaling pathways and enhanced cell proliferation [49]. In the current study, tuber hexose concentrations tended to increase in the 25 °C/Elev_CO_2__ treatment while sucrose was unaffected (Figure 6). Invertase activities were increased in response to Elev_CO_2__ (Figure 7), consistent with potential involvement in increasing hexose levels and sugar signaling through a hexose signaling pathway [50]. In tomato (*Solanum lycopersicum*) fruit, evidence from gene silencing indicates that cell wall invertase is essential for sugar signaling and development [51]. While sugar signaling has been studied with respect to potato tuber carbon flux and starch synthesis, it merits further attention with respect to regulation of cell proliferation.

Contrary to the general pattern with Elev_CO_2__, in the high temperature treatments, at both tuber initiation and tuber bulking, partial amelioration provided by elevated CO_2_ was substantially due to enhanced cell expansion in the high temperature treatments, whereas cell expansion was not involved in the normal temperature treatments (Figure 4 and Figure 5). In studies of the effects of shade on tuber development, the extent of endoreduplication usually increased where average cell volume was higher [40]. However, in the current study, even though high temperature enhanced cell expansion, it did not affect endoreduplication (Figure 4 and Figure 5). Endoreduplication is common in plants, and in the present study a high proportion of cells were endoreduplicated to nuclear DNA contents ≥ 8C; they were 35 and 49% at tuber initiation and tuber bulking, respectively. The roles of endoreduplication are still a matter of speculation, though in other plant systems it has been suggested that endoreduplication has a role in stress response [52]. In the present system there was no evidence for an endoreduplication response to Elev_CO_2__ or high temperature. So, the mechanism by which environment affects tuber growth and development may be different from that involved in other systems.

### 3.4. Global Climate Change

In relation to global climate change, the current study provides insight into potato’s sensitivity to various combinations of CO_2_ and temperature at two discrete developmental phases. In the field, although elevated CO_2_ is a constant condition throughout a crop season, high temperature stress often occurs for relatively short periods of time, such as one or several days. Given the short time over which heat stress episodes commonly occur in the field, it is of interest to determine whether there are particular stages of potato development that are more sensitive to heat stress. Evaluations of simulation models for potato agronomic responses and global climate change indicate that model variation is modest for predicted CO_2_ but is particularly uncertain for temperature [5]. This suggests that more knowledge about the nature of heat susceptibility in potato would be valuable in guiding future efforts to improve the crop genetically and to optimize management [12,21,53]. In regard to whole plant DMAR, the current studies indicate that the tuber initiation stage may be somewhat more sensitive to stress than tuber bulking. This may be a consequence of the considerably greater sensitivity of cell proliferation and organelle developmental processes at tuber initiation, whereas starch accumulating cells at tuber bulking may be more robust and less responsive to stress. Moreover, plants have numerous feedbacks in their regulatory systems such that changes in tuber sink capacity can provide feedback regulation of photosynthesis [25].

## 4. Materials and Methods

### 4.1. Plant Material

Potato plants (cv. Katahdin) were grown from tuber cuttings in a greenhouse with 14 h supplemental lighting and were watered with nutrient solution (Peter’s 15-16-17 fertilizer, W.R. Grace and Co., Fogelsville, PA, USA) which supplied the following elemental nutrients: 33 mg L^−1^ of NO_3_-N, 86 mg L^−1^ of NH_4_-N, 42 mg L^−1^ of urea-N, 74 mg L^−1^ of P, 151 mg L^−1^ of K. Pots (6 L) contained peat, vermiculite, and perlite (1:1:1 *v*/*v*/*v*) with 3 g of pulverized limestone, 17 g of CaSO_4_, 21 g of powdered FeSO_4_, 0.5 g of fritted trace elements (Peters FTE 555, Scotts Co., Marysville, OH, USA), and 1.5 g of wetting agent (AquaGro G, Aquatrols, Pennsauken, NJ, USA). After two weeks, young plants were trimmed to include one shoot and re-sown in new 12 L pots with one plant/pot. After transplanting, plants were grown in the greenhouse for an additional 4 weeks, then the plants were transferred to four matched growth chambers where short days (10 h photoperiod) were imposed to initiate tuber formation, as described below. Nutrient solution and watering regime were the same as in the greenhouse.

On the day that plants were transferred to growth chambers for imposition of temperature × CO_2_ treatments, plants from a set of uniform material were randomly assigned to the treatments, as described below; concurrently, a subset was designated for immediate harvest at time = 0 for estimating the dry matter of plants parts at the onset of treatments.

### 4.2. Temperature and CO_2_ Control

Controlled-environment chambers (Model CEL-63-10, Sherer Inc, Marschall, MI, USA) had interior dimension of 112 × 74 cm (width × depth) and 600 μmol photons (photosynthetically active radiation, 400–700 nm) m^−2^ s^−1^ at the top of the canopy, supplied by fluorescent lamps. Temperature regime was 25/18 °C (day/night) in the normal temperature treatment and 35/25 °C (day/night) in the high temperature treatment. CO_2_ treatments were either 700 μmol CO_2_ mol^−1^ air (elevated CO_2_) or between 350 and 400 μmol CO_2_ mol^−1^ air (low CO_2_). Chamber CO_2_ concentration was monitored with a calibrated infrared gas analyzer [30]. The four growth chambers were used to impose a 2 × 2 matrix of temperature × CO_2_ treatments at two stages of development: (1) during the first 2 weeks after switch from 14 to 10 h days (weeks after short d; WASD), which stimulates tuber production [hereinafter described as the tuber initiation stage (TI)], or (2) during the next two weeks after switch to short days, when starch accumulation predominates [hereinafter described as the tuber bulking (TB) stage]. Plants for the tuber bulking treatments were first given a short-day (10 h photoperiod) treatment for two weeks in chambers at the normal conditions of 25/18 °C (day/night) and low CO_2_.

### 4.3. Tuber Sampling Method

To representatively sample tubers for analyses, all tubers within each plant were characterized into three groups by size, each group containing about one third of the tuber fresh biomass in the entire plant. The grouping started by ranking tubers within a plant by size and, starting with the largest tuber, assigning it to the largest size-category, then continuing to the next smaller tuber until the sum of tuber fresh biomass in the category accounted for about one third of the tuber biomass of the entire plant. In this way, at least two tubers were chosen for this size category. Then tubers were assigned to the next size category until the summed biomass in each of the three categories accounted for one third of the total. In each category, three tubers (or two, depending on availability) were randomly chosen for sugar levels or enzymes activities determination.

### 4.4. Assay Methods

From representative tubers, two cores, longitudinal and transverse, were immediately withdrawn with a 0.5 cm diameter cylindrical borer and fixed in ethanol/acetic acid (3:1) solution for flow cytometry. Then the apical-bud end (rose end) of the tuber was cut off 1 cm proximal from the apex, and a 0.5 cm thick slice was obtained from the apical end, frozen in liquid nitrogen, and stored at −20 °C for further determination of sugars and related enzyme activities. Leaves, stems, and remaining portions of tubers were dried at 50 °C to constant weight and weighed. Tuber dry weights were corrected for the fresh weight removed for cell cytometry and enzyme activity.

For flow cytometry analysis, core slices were digested with 0.5% (*w*/*v*) pectinase (EC 3.2.1.15, MP Biomedicals, Solon, OH, USA) for 18 h without shaking at 37 °C, then cooled in a 4 °C refrigerator overnight before mechanically disrupting cell walls and releasing nuclei into the medium. After about 18 h of shaking (150 rpm) the tubers were stored in a 4 °C refrigerator then filtered through nylon-mesh fabric with 100 μm openings (Nitex, Teco Inc, Briarcliff Manor, NY, USA). The DNA-binding fluorochrome propidium iodide was added to the filtrate to a final concentration of 90 μM in a 10 mM Tris-HCl buffer (pH 7.4). A 100 μL aliquot of filtrate was counted by flow cytometry using either a FACScan analyzer (Becton Dickinson, Mountain View, CA, USA), or an Epic Profile (Coulter Electronics, Hialeah, FL, USA) operating with an argon-neon laser (488 nm).

For determination of sugars and related enzymes, 0.5 cm diameter disks were cut out from the zone rich in vascular bundles in the perimedullary zone near the perimedulla/cortex interface of slices with a cylindrical borer and then homogenized with 50 mM Hepes-KOH (pH 7.4) buffer (tissue:buffer 1:2(*w*/*v*)) containing 5 mM MgCl_2_, 1 mM EGTA, 1 mM EDTA, 40% (*v*/*v*) glycerol, 0.1% bovine serum albumin, 0.5 mM dithiotheritol and 2% insoluble polyvinylpyrrolidone and stored at −20 °C. Ten microliter aliquots of supernatant were mixed with 90 μL ethanol and stored for sugar assay. The concentrations of glucose, fructose and sucrose were determined using an enzyme-coupled assay based on hexokinase (EC 2.7.1.1, 0.14 unit) and glucose-6-phosphate dehydrogenase (EC 1.1.1.49, 0.07 unit), and using phosphoglucoisomerase (EC 5.3.1.9, 0.2 unit) and invertase (EC 3.2.1.26, 80 units) for fructose and sucrose interconversions [54].

Acid invertase activities were determined in the sucrose hydrolysis direction. For soluble acid invertase, 0.5 mL of buffer-extract was centrifuged at 16,000× *g* and desalted on a Sephadex G-25M to obtain enzyme extract. For cell-wall-bound acid invertase, 0.5 mL of buffer-extract was centrifuged at 16,000× *g* and, then, the pellet was resuspended in 1 mL of 1 M NaCl and incubated at 4 °C at least 12 h for salt extraction. After centrifugation at 16,000× *g*, the salt extract was desalted on a Sephadex G-25M to obtained enzyme extract. Both soluble and cell-wall-bond acid invertase activities were determined by mixing enzyme extract (150 μL) with 50 μL of 1 M sucrose to start the enzyme reaction. The reaction was incubated for 75 min at 24 °C and the glucose + fructose produced was assayed as before [54]. All enzyme assays were linear with time and the amount of enzyme extract added.

### 4.5. Data Analysis

The experiments were conducted in three batches with two plants in each (6 biological replicates). A plant was considered an experimental unit. Each batch contained a complete set of treatments, which were randomly assigned to four matched chambers set at the two CO_2_ concentrations and temperatures (a 2 × 2 set of treatments), as described above. Data were modeled using a simple linear model with batch, temperature treatment (T), CO_2_ treatment, and T × CO_2_ interaction as sources of variation. Analysis of variance was conducted in R version 3.6.0 [55] using the *lm* function. Tukey HSD multiple-range significant-difference tests were used for multiple comparisons.

## 5. Conclusions

The current study elucidated the effects of elevated CO_2_ and high temperature on potato tuber development at two stages: tuber initiation and tuber bulking. While whole-plant dry matter accumulation rate was increased by elevated CO_2_ about 30% at both stages, the responses of plant parts differed considerably. At the tuber initiation stage, and at normal day-time temperature of 25 °C, elevated CO_2_ increased tuber dry matter accumulation rate by more than 60%, while leaf and stem DMAR was not affected, even though these organs collectively represented almost two thirds of the sink-organ DMAR. At the tuber bulking phase, when most of plant photosynthate is used for starch storage in tubers, leaves had very low DMAR and stems had a net loss in dry matter, so almost all the benefit from elevated CO_2_ was obtained by tubers. The extent to which high temperature stress affected plant growth and sink development differed considerably between stages. Whole-plant dry matter accumulation rate, was inhibited by high temperature treatment about twice as much at the stage of tuber initiation than at tuber bulking. In contrast to CO_2_ effects at 25 °C, where tubers primarily benefited, at high temperature stress, elevated CO_2_ benefitted leaf and stem growth in addition to tuber growth, and hence partially ameliorated high temperature inhibition. This suggests that growing plant parts have a development window within which they are capable of responding to improved photosynthate supply. At the tuber initiation stage, with 25 °C, elevated CO_2_ had its primary effect on cell proliferation such that tuber cell numbers in the elevated CO_2_ treatment were 62% greater than with the corresponding low CO_2_ control. In contrast, cell volume and the proportion of cells that had undergone endoreduplication were unaffected. These findings indicate that the tuber initiation stage and cell division process are particularly responsive to elevated CO_2_ and high temperature stress. To fully understand impacts of climate change and to develop improved crops, attention should be paid to the timing of high-temperature stress episodes with respect to this stage.

## Figures and Tables

**Table 1:** Total plant dry-matter accumulation rate during temperature and CO_2_ treatments at the tuber initiation or tuber bulking stage. CO_2_ treatments were Low and Elevated (Elev). The pooled standard error of the design (SE) are shown (*n* = 6); values labeled with different letters are significantly (*p* ≤ 0.05) different.

		Stage of Development			
Treatment		When Treatment Was Imposed			
temperature	atm CO_2_	Tuber Initiation		Tuber Bulking	
		dry-matter accumulation rate			
°C	[CO_2_]	g DW plant^−1^ wk^−1^			
25	Elev	24.3	c	37.7	c
25	Low	18.9	cb	28.0	b
35	Elev	15.8	ba	31.9	cb
35	Low	8.3	a	19.0	a
	SE	1.6		1.8	

## Data Availability

The data presented in this study are available on request from the corresponding author.

## Supplementary Materials

The following are available online at https://www.mdpi.com/article/10.3390/plants10050871/s1, Suppl. Figure S1. Distribution of whole-plant tuber production among individual tuber size-classes in plants exposed to temperature (25 or 35 °C) or CO_2_ treatments (Low_CO_2__ or Elev_CO_2__). (a) Tuber initiation stage; (b) tuber bulking stage. Plants were harvested after exposure to treatment for 2 weeks. Tubers exceeding 1 cm diameter were counted. Tuber production was the sum of tuber fresh weight within the defined size-range category. Data was from 6 replicate plants per stage of development.
