# Supplementary figures and images for: Role of Tuber Developmental Processes in Response of Potato to High Temperature and Elevated CO2
Source: Plants (Basel). 2021 Apr 26;10(5):871. doi: 10.3390/plants10050871 (PMC8146319; doi:10.3390/plants10050871)

**a**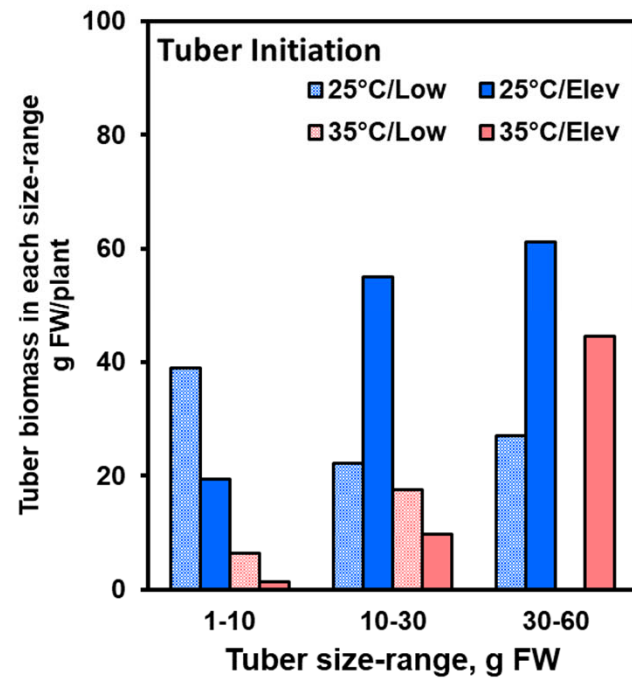**b**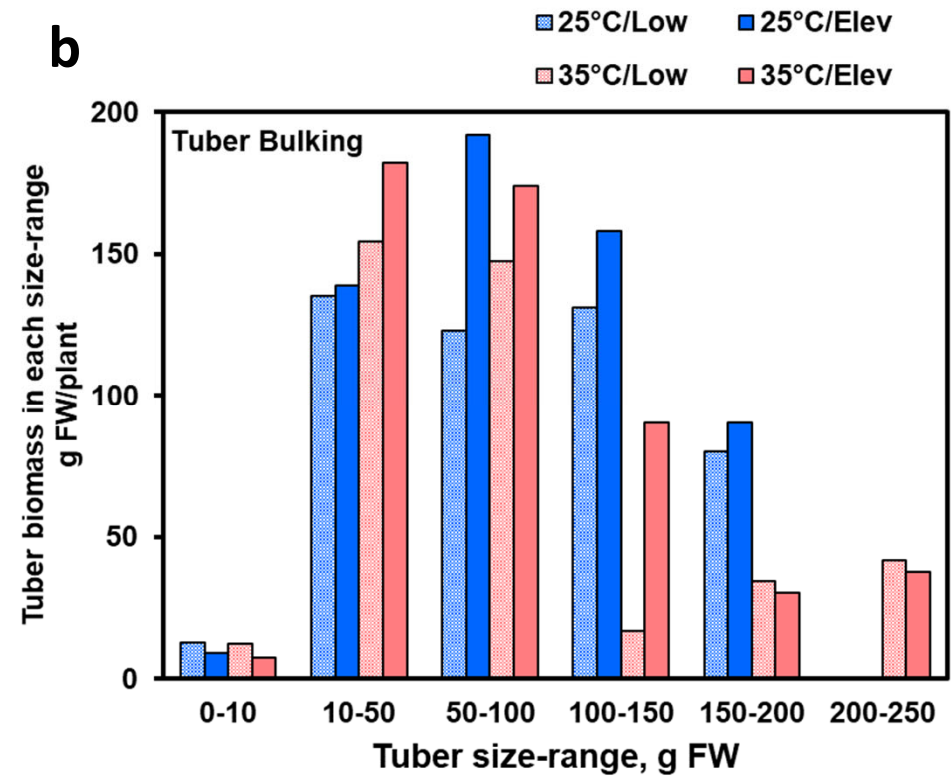

Suppl. Figure S1

Supplement: Supplementary file 1 [file plants-10-00871-s001.zip › plants-1199264-SI.pdf]
